# Early systemic sclerosis: marker autoantibodies and videocapillaroscopy patterns are each associated with distinct clinical, functional and cellular activation markers

**DOI:** 10.1186/ar4236

**Published:** 2013-05-29

**Authors:** Gabriele Valentini, Antonella Marcoccia, Giovanna Cuomo, Serena Vettori, Michele Iudici, Francesco Bondanini, Carlo Santoriello, Aldo Ciani, Domenico Cozzolino, Giovanni Maria De Matteis, Salvatore Cappabianca, Filiberto Vitelli, Alberto Spanò

**Affiliations:** 1 Rheumatology Unit, Second University of Naples, via Pansini 5, 80131 Naples, Italy; 2 Angiology Unit, Sandro Pertini Hospital, via dei Monti Tiburtini 385, 00157 Rome, Italy; 3 Clinical Biochemistry Service, Sandro Pertini Hospital, via dei Monti Tiburtini 385, 00157 Rome, Italy; 4 Respiratory Physiopathology Unit, ASL-SA1, via Santoriello 2, Cava dei Tirreni (SA), Italy; 5 Pneumology Unit, Sandro Pertini Hospital, via dei Monti Tiburtini 385, 00157 Rome, Italy; 6 Internal Medicine Unit, Second University of Naples, via Pansini 5, 80131 Naples, Italy; 7 Cardiology Unit, Sandro Pertini Hospital, via dei Monti Tiburtini 385, 00157 Rome, Italy; 8 Radiology, Radiotherapy and Nuclear Medicine Unit, Second University of Naples, Piazza Miraglia 5, 80131 Naples, Italy; 9 Radiology Unit, Sandro Pertini Hospital, via dei Monti Tiburtini 385, 00157 Rome, Italy

**Keywords:** Raynaud's phenomenon, early systemic sclerosis, systemic sclerosis marker autoantibodies, nailfold videocapillaroscopy, preclinical organ involvement, puffy fingerscirculating activation markers, carboxyterminal propeptide of collagen I, soluble E-selectin, soluble IL-2 receptor alpha

## Abstract

**Introduction:**

Early systemic sclerosis (SSc) is characterized by Raynaud's phenomenon together with scleroderma marker autoantibodies and/or a scleroderma pattern at capillaroscopy and no other distinctive feature of SSc. Patients presenting with marker autoantibodies plus a capillaroscopic scleroderma pattern seem to evolve into definite SSc more frequently than patients with either feature. Whether early SSc patients with only marker autoantibodies or capillaroscopic positivity differ in any aspect at presentation is unclear.

**Methods:**

Seventy-one consecutive early SSc patients were investigated for preclinical cardiopulmonary alterations. Out of these, 44 patients and 25 controls affected by osteoarthritis or primary fibromyalgia syndrome were also investigated for serum markers of fibroblast (carboxyterminal propeptide of collagen I), endothelial (soluble E-selectin) and T-cell (soluble IL-2 receptor alpha) activation.

**Results:**

Thirty-two of the 71 patients (45.1%) had both a marker autoantibody and a capillaroscopic scleroderma pattern (subset 1), 16 patients (22.5%) had only a marker autoantibody (subset 2), and 23 patients (32.4%) had only a capillaroscopic scleroderma pattern (subset 3). Patients with marker autoantibodies (*n *= 48, 67.6%) had a higher prevalence of impaired diffusing lung capacity for carbon monoxide (*P *= 0.0217) and increased serum levels of carboxyterminal propeptide of collagen I (*P *= 0.0037), regardless of capillaroscopic alterations. Patients with a capillaroscopic scleroderma pattern (*n *= 55, 77.5%) had a higher prevalence of puffy fingers (*P *= 0.0001) and increased serum levels of soluble E-selectin (*P *= 0.0003) regardless of marker autoantibodies.

**Conclusion:**

These results suggest that the autoantibody and microvascular patterns in early SSc may each be related to different clinical-preclinical features and circulating activation markers at presentation. Longitudinal studies are warranted to investigate whether these subsets undergo a different disease course over time.

## Introduction

Early systemic sclerosis (SSc) is a condition characterized by Raynaud's phenomenon (RP) associated with SSc marker autoantibodies (anti-Scl-70, anticentromere antibodies, anti-RNA polymerase III, anti-fibrillarin, anti-PmScl, and anti-Th/To) and/or nailfold videocapillaroscopy (NVC) findings typical of SSc (namely, megacapillaries and avascular areas - commonly referred to as an 'NVC scleroderma pattern'). By definition, patients with early SSc do not show any distinctive clinical manifestation of the disease (namely, sclerodactyly, digital ulcers/scars, two or more teleangectasias, clinically visible nailfold capillaries, cutaneous calcinosis, X-ray bibasilar lung fibrosis, X-ray esophageal dysmotility, electrocardiographic signs of myocardial fibrosis, or a serum creatinine increase suggestive of scleroderma renal crisis) except puffy fingers and/or arthritis [1-4].

Various attempts have been made to find a correct classification for these patients who do not meet the preliminary 1990 American College of Rheumatology (ACR) SSc criteria [5] in order to foster their inclusion in clinical trials. In fact, in 2001 LeRoy and Medsger proposed that RP patients with SSc marker autoantibodies or an NVC scleroderma pattern should be classified as being affected by limited SSc [1]. Seven years later, Koenig and colleagues validated the LeRoy and Medsger criteria in a large 20-year prospective study [2]. They found that, at the last follow-up, patients presenting with SSc marker autoantibodies and a scleroderma pattern at capillaroscopy (referred to as subset 1 in the present paper), with no other manifestation distinctive of the disease other than puffy fingers and/or arthritis, had developed definite SSc 60 times more frequently than patients presenting with only RP. Patients with either only SSc marker autoantibodies (subset 2) or a capillaroscopic scleroderma pattern (subset 3) had developed definite SSc five and eight times more frequently, respectively, than patients presenting with only RP. Koenig and colleagues therefore suggested that such cases be labeled collectively as early SSc.

Two years ago, we reported that 42% of thus defined early SSc patients admitted to our tertiary Rheumatology Unit had preclinical functional alterations of the esophagus, lung or heart [3]. Subsequently, we showed that a high percentage of early SSc patients develop definite SSc within 5 years of presentation and found that some circulating fibroblast, endothelial and T-cell activation markers could be used as predictors of disease evolution besides marker autoantibodies and NVC findings [4]. In their study, Koenig and colleagues did not systematically investigate visceral involvement and neither did they consider circulating activation markers [2]. In our previous papers we were unable to try any subsetting among our early SSc patients because of the low number of early SSc patients with only an NVC scleroderma pattern and negative for SSc marker autoantibodies admitted to our unit [3,4].

In the present study, we evaluated whether clinical, preclinical functional or cellular activation markers are related to distinct early SSc subsets at presentation by merging our early SSc cohort with that recruited at a secondary Angiology Unit, where a significant number of patients with early SSc were SSc marker antibody-negative and capillaroscopy-positive.

## Materials and methods

### Patients

Patients admitted for evaluation of RP from 1 November 2000 to 30 June 2012 to the outpatient clinic of the Rheumatology Unit of the Second University of Naples and from 1 January 2008 to 30 June 2012 to the outpatient clinic of the Angiology Unit of the Pertini Hospital in Rome who met the Koenig and colleagues criteria for early SSc (that is, RP with SSc marker autoantibodies and/or an NVC scleroderma pattern without any clinical manifestation of definite SSc) [2] were enrolled in the study after giving written informed consent.

The diagnosis of RP was confirmed if patients fulfilled LeRoy and Medsger's criteria [6]; that is, if a cold challenge induced bilateral, episodic biphasic or triphasic (pallor followed by dusky blueness and/or redness) color changes of fingers. According to standard clinical practice, and for the purpose of a correct disease classification, patients underwent a complete screening workplan.

### Routine assessment

Each patient was investigated to identify any feature that precluded inclusion in the study. In detail, we looked for the following features: dysphagia, effort dyspnea, and findings consistent with previous scleroderma renal crisis at history; symmetrical skin sclerosis, digital ulcers/scars, two or more teleangectasias, clinically visible nailfold capillaries, cutaneous calcinosis, and recent-onset accelerated or malignant arterial hypertension at physical examination; bibasilar lung fibrosis at chest X-ray scan; esophageal dysmotility at barium esophageal X-ray scan; and blocks and/or Q waves at electrocardiography. In addition, blood cell count, urinalysis, blood urea nitrogen, serum creatinine, alanine aminotransferase, aspartate aminotransferase, erythrosedimentation rate, serum protein electrophoresis with the evaluation of γ-globulin concentration, and serum C3 and C4 concentration were also evaluated.

### Nailfold videocapillaroscopy assessment

NVC was carried out with an optical probe videocapillaroscope equipped with a ×200 magnification contact lens and connected to image analysis software (Videocap; DS MediGroup, Milan, Italy). The nailfold of the second, third, fourth and fifth fingers was examined bilaterally in each patient. Four consecutive fields extending over 1 mm in the middle of the nailfold were studied per finger. All images of patients from both centers were stored and reviewed by a physician (MI) experienced in NVC [3,4,7]. The degree of capillary enlargement (on a scale of 0 to 3) and capillary loss (graded A to D) were investigated. Megacapillaries (capillary enlargement ≥2) and/or avascular areas (capillary loss grade ≥C) were considered a scleroderma pattern [8,9]. Moreover, since NVC active and late patterns, which are characterized by a lower capillary density, have been found to be associated with more severe disease in SSc patients [10], we also subdivided patients according to the mean capillary number per millimeter; that is, >9/mm, 9 to 7/mm, 6 to 4/mm, and ≤3/mm.

### Autoantibody profile assessment

An autoantibody screening and profiling of sera collected at the first visit was performed. Antinuclear antibodies and anticentromere antibodies were searched for by a conventional indirect immunofluorescence assay on HEp2 cells, using fluorescent human γ-globulins as detection antibodies and a serum dilution of 1:160 as the cutoff value (Astra srl, Pavone Canavese, Italy). Anti-double-stranded DNA antibodies were also searched for by indirect immunofluorescence on Crithidia Luciliae, using fluorescent human γ-globulins as detection antibodies and a serum dilution of 1:40 as the cutoff value (Astra srl). Anti-Scl-70, anti-PmScl, anti-SSA, anti-SSB, anti-Sm, anti-Jo1, and anti-U1RNP antibodies were identified by commercially available ELISA kits (Chematil srl, Angri, Italy), using 25 U/ml as the cutoff value. Anti-RNA polymerase III and anti-fibrillarin antibodies were identified by EliA Varelisa test (Phadia, Freiburg, Germany), using 150 U/ml as the cutoff value. Anti Th/To were identified by western blotting (reagents by Arnika srl, Milano, Italy). To avoid a differential verification bias, sera were blindly exchanged between the two centers and only confirmed results were considered. Each result was confirmed.

### Assessment of preclinical internal organ involvement

Patients from both centers underwent B-mode echocardiography and lung function study. Echocardiographic examination was performed as reported elsewhere [11]. The detection of diastolic abnormalities at B-mode echocardiography, indicated by an inverted ratio between early/(atrial) late ventricular filling velocity (E/A ratio <1), in the absence of arterial hypertension, coronary artery disease and other symptoms/signs of cardiac disease, was regarded as early scleroderma heart involvement [12]. The detection of a diffusing lung capacity for carbon monoxide (DLCO) or a forced vital capacity <80% of the predicted values in the absence of a smoking habit and/or obstructive lung disease at lung function study was regarded as SSc lung involvement [13,14]. Moreover, after further informed consent, patients from the Rheumatology Unit with a reduced DLCO underwent high-resolution computed tomography of the chest [15].

### Fibroblast, endothelial and T-cell activation markers

Early SSc patients for whom a baseline serum specimen was available and 25 controls, matched for sex and age and affected by osteoarthritis or primary fibromyalgia syndrome, were investigated at the Rheumatology Unit laboratory for the fibroblast, endothelial and T-cell activation markers carboxyterminal telopeptide of type I collagen (ICTP), soluble E-selectin (sE-selectin) and soluble IL-2 receptor alpha (sIL-2Rα), as described previously [4]. Briefly, sIL-2Rα and sE-selectin concentrations were measured by a multiplex suspension immunoassay (expressed as pg/ml), based on the use of spectrally encoded beads, each coupled with a capture antibody specific to the analyte of interest, as the solid support and a biotinylated detection antibody-streptavidine-phycoerythrin complex as the reporter system (all reagents were from Merk Millipore, Billerica, MA, USA) to be read by a double laser-based fluorimetric instrument (Luminex 200; Luminex Corporation, Austin, TX, USA). The ICTP concentrations were measured by a conventional competitive radioimmune assay (expressed as µg/l) (UniQ kits; Orion Diagnostica, Espoo, Finland). We did not measure the aminoterminal propeptide of type III collagen serum concentration because in our previous study it did not differ between early SSc and either undifferentiated connective tissue disease patients or controls at baseline [4].

Based on the autoantibody and capillaroscopic profiles, early SSc patients were divided into three subsets: subset 1, patients with both SSc marker autoantibody positivity and an NVC scleroderma pattern; subset 2, patients with only SSc marker autoantibody positivity; and subset 3, patients with only an NVC scleroderma pattern.

The study was reviewed and approved by the Azienda Ospedaliera Universitaria Seconda Università degli Studi di Napoli Ethics Committee and by the ASL RM/B Ethics Committee.

### Statistical analysis

GraphPad Prism software version 6.0 (GraphPad Software Inc., San Diego, CA, USA) was used for statistical analysis. Continuous data were expressed as the mean ± standard deviation and the median with range, and were compared by the unpaired Student's *t *test or Mann-Whitney U test as appropriate when two groups were analyzed, and by one-way analysis of variance or Kruskal-Wallis test as appropriate when three or more groups were compared. Categorical data were analyzed by Fisher's exact test and the chi-square test when two or three groups, respectively, were considered.

## Results

Forty-five patients were enrolled at the Rheumatology Unit of the Second University of Naples and 26 patients at the Angiology Unit of the Pertini Hospital in Rome. Table 1 presents the main epidemiologic, laboratory and capillaroscopic features of the patients enrolled by the Rheumatology and Angiology Units. There was no difference between the two series in terms of age, sex and time interval elapsed from the onset of RP. However, the prevalence of subset 1 patients was significantly higher (26/45 (57.8%) vs. 6/26 (23.1%), *P *= 0.0098) and the prevalence of subset 3 patients significantly lower (3/45 (6.7%) vs. 20/26 (76.9%), *P *= 0.0001) in the Rheumatology series. Subset 2 patients were recruited only by the Rheumatology Unit.

**Table 1:** Epidemiologic, laboratory and capillaroscopic features of the two early systemic sclerosis series

Feature	Rheumatology Unit (*n *= 45)	Angiology Unit (*n *= 26)	*P *value
Sex (female/male ratio)	43/2	22/4	NS
Age (years)	41 (17-73)	37.5 (16-71)	NS
RP duration (years)	3 (0.5-24)	3 (1-20)	NS
Subset 1: antibody-positive, NVC-positive	26 (57.8)	6 (2.2)	0.0098
Anti-Scl-70 antibody-positive	5 (19.2)	0	
ACA-positive	19 (73.1)	3 (50)	
Anti-RNA polymerase III antibody-positive	2 (7.7)	3 (50)	
Megacapillaries only	21 (80.8)	3 (50)	
Avascular areas ± megacapillaries	5 (19.2)	3 (50)	
Subset 2: antibody-positive, NVC-negative	16 (35.6)	0	-
AntiScl-70 antibody-positive	4 (25)	0	
ACA-positive	12 (75)	0	
Anti-RNA polymerase III antibody-positive	0	0	
Subset 3: antibody-negative, NVC-positive	3 (6.7)	20 (76.9)	0.0001
Megacapillaries only	3 (100)	20 (100)	-
Avascular areas ± megacapillaries	0	0	-

All data expressed as median (range) or number (percentage). ACA, anticentromere antibody; NVC, nailfold videocapillaroscopy; RP, Raynaud's phenomenon.

By definition, no patient presented any clinical feature of SSc other than puffy fingers or arthritis. The prevalence of puffy fingers was significantly higher in both subset 1 and subset 3 Angiology Unit patients than in the respective Rheumatology Unit patients (6/6 vs. 4/26, *P *= 0.004 for subset 1; 19/20 vs. 1/3, *P *= 0.04 for subset 3). The prevalence of arthritis did not differ statistically between the two centers (1/26 (3.85%) for Rheumatology Unit patients vs. 2/6 (33.3%) for Angiology Unit patients, *P *>0.05 for subset 1; 0/3 (0%) for Rheumatology Unit patients vs. 2/20 (10%) for Angiology Unit patients, *P *>0.05 for subset 3). Because of the low number of subset 3 patients recruited at the Rheumatology Unit and of subset 1 patients admitted to the Angiology Unit, in order to increase the power of the analysis we merged the two series.

**Figure 1:**
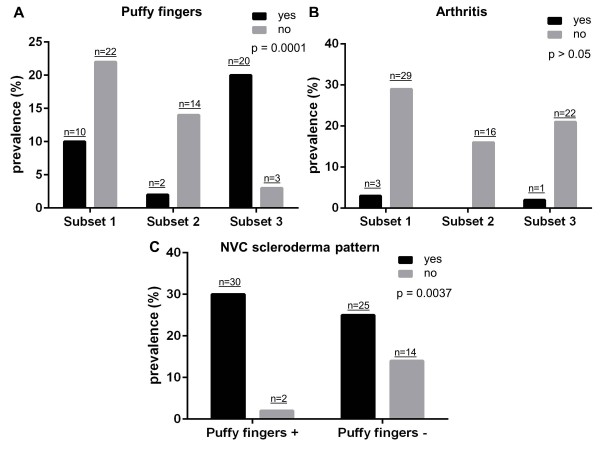
**Prevalence of puffy fingers, arthritis, and nailfold videocapillaroscopy scleroderma pattern in early systemic sclerosis patients**. Prevalence of **(A) **puffy fingers and **(B) **arthritis in the 71 early systemic sclerosis (SSc) patients from both centers, divided into three subsets according to the SSc marker autoantibody status and the nailfold videocapillaroscopy (NVC) pattern: presence of both SSc marker autoantibodies and NVC scleroderma pattern (subset 1), presence of SSc marker autoantibodies only (subset 2), and presence of NVC scleroderma pattern only (subset 3). **(C) **Prevalence of NVC scleroderma pattern in all patients from both centers divided according to the presence/absence of puffy fingers. Chi-square test was applied for statistical analysis of data reported in (A) and (B); Fisher's exact test was applied for statistical analysis of data reported in (C).

Figure 1 shows the prevalence of puffy fingers (Figure 1A) and arthritis (Figure 1B) in the three subsets. The prevalence of puffy fingers was significantly higher in subset 3 patients (20/23, 87%) from the pooled series than in subset 1 (10/32, 31.3%) and subset 2 (2/16, 12.5%) patients (*P *= 0.0001) (Figure 1A). Thirty out of the 32 (93.75%) patients across the three subsets with puffy fingers had a positive NVC, and 25/39 (64.1%) patients without puffy fingers had a positive NVC (*P *= 0.0037) (Figure 1C).

**Figure 2:**
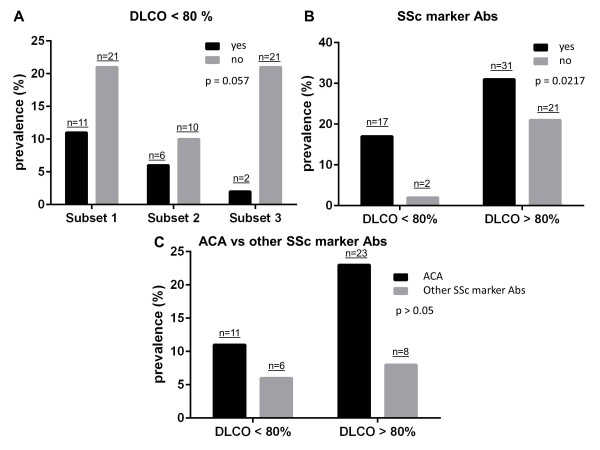
**Prevalence of diffusing lung capacity for carbon monoxide impairment and systemic sclerosis marker autoantibody positivity**. **(A) **Prevalence of diffusing lung capacity for carbon monoxide (DLCO) impairment (<80% of the predicted value) in the 71 early systemic sclerosis (SSc) patients from both centers, divided into three subsets according to the SSc marker autoantibody status and the nailfold videocapillaroscopy (NVC) pattern: presence of both SSc marker autoantibodies and NVC scleroderma pattern (subset 1), presence of SSc marker autoantibodies only (subset 2), and presence of NVC scleroderma pattern only (subset 3). **(B) **Prevalence of general SSc marker autoantibody positivity and **(C) **prevalence of either anticentromere antibody (ACA) or other SSc marker autoantibody positivity in all patients from both centers divided according to the presence/absence of DLCO impairment. Chi-square test was applied for statistical analysis of data reported in (A). Fisher's exact test was applied for statistical analyses of data reported in (B) and (C). Abs, autoantibodies.

The prevalence of functional abnormalities did not differ between each subset of patients from the two centers. A forced vital capacity <80% of the predicted value and an inverted E/A ratio were found only in 2/32 (6.25%) and 1/32 (3.13%) subset 1 patients, respectively, and in no subset 2 and subset 3 patients. However, an impaired DLCO was more prevalent in subset 1 (11/32, 34.4%) and subset 2 (6/16, 37.5%) patients than in subset 3 (2/23, 8.7%) patients (*P *= 0.057) (Figure 2A). In addition, out of the 48 marker autoantibody-positive patients (that is, subset 1 + subset 2), DLCO <80% of the predicted value was detected in 17 (35.4%) versus 2/23 (8.7%) marker autoantibody-negative subset 3 patients (*P *= 0.0217) (Figure 2B). Lastly, 6/14 (42.9%) patients with SSc marker autoantibodies other than anticentromere antibodies (three anti-Scl-70; three anti-RNA polymerase III) had a reduced DLCO as compared with 11/34 (34.4%) anticentromere antibody-positive patients. The difference did not reach statistical significance (Figure 2C).

A ground glass opacity involving <5% of inferior lobes and an interlobular septal thickening were found in two patients and two different patients, respectively, by lung high-resolution computed tomography.

**Figure 3:**
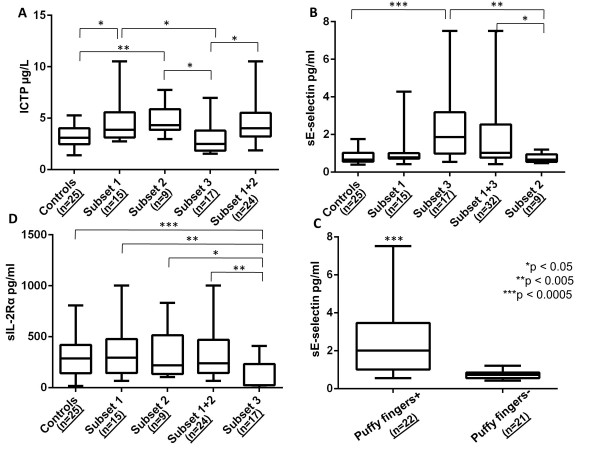
**Serum levels of fibroblast, endothelial, and T-cell activation markers**. Serum levels of **(A) **fibroblast (carboxyterminal telopeptide of type I collagen (ICTP)), **(B) **endothelial (soluble E-selectin (sE-selectin)), and **(D) **T-cell (soluble IL-2 receptor alpha (sIL2Rαa)) activation markers in 25 controls affected by ostheoarthritis or fibromyalgia, and 44 early systemic sclerosis (SSc) patients, divided into three subsets according to the SSc marker autoantibody status and the nailfold videocapillaroscopy (NVC) pattern: presence of both SSc marker autoantibodies and NVC scleroderma pattern (subset 1), presence of SSc marker autoantibodies only (subset 2), and presence of NVC scleroderma pattern only (subset 3). **(C) **Serum levels of sE-selectin in all patients from both centers divided according to the presence/absence of puffy fingers. Kruskal-Wallis test and Mann-Whitney U test were applied for statistical analysis of data reported in (A), (B), and (D). Mann-Whitney U test was applied for statistical analysis of data reported in (C).

Sera collected at admission were available for 22/45 (48.9%) patients from the Rheumatology Unit and for 22/26 (84.6%) patients from the Angiology Unit. The clinical, serological and NVC features of the 22 Rheumatology Unit patients reflected the characteristics of the whole series of the center (data not shown). Figure 3 shows serum levels of the investigated activation markers in the three early SSc subsets and in controls. Two main findings emerged from this analysis. Firstly, serum concentrations of ICTP were significantly higher in patients with SSc marker autoantibodies (median = 4.025 µg/l in subset 1 + subset 2, range 1.883 to 10.53 µg/l) than in subset 3 patients (median = 2.51 µg/l, range 1.55 to 6.972 µg/l; *P *= 0.0108) (Figure 3A). In detail, 4/15 (26.7%) subset 1 patients and 3/9 (33.3%) subset 2 patients had ICTP levels that exceeded the 95th percentile (5.05 µg/l) of the values measured in controls with respect to 2/17 (11.8%) subset 3 patients. Secondly, levels of sE-selectin were significantly higher in patients with an NVC scleroderma pattern (median = 1.031 pg/ml in subset 1 + subset 3, range 0.425 to 7.51 pg/ml) than in subset 2 patients with only SSc marker autoantibodies (median = 0.67 pg/ml, range 0.486 to 1.203 pg/ml; *P *= 0.033) (Figure 3B). In detail, sE-selectin levels exceeded the 95th percentile of the values recorded in controls (1.709 pg/ml), in 11/17 (64.7%) subset 3 patients, in 2/15 (13.3%) subset 1 patients and in 0/9 subset 2 patients (*P *= 0.0003). Noteworthy, we found that among all three subsets sE-selectin levels were significantly higher in patients with puffy fingers than in those without puffy fingers (2.013 pg/ml, range 0.55 to 7.51 vs. 0.725 pg/ml, range 0.425 to 1.203 pg/ml; *P *< 0.0001) (Figure 3C). Finally, sIL-2Rα levels were lower in SSc marker autoantibody-negative patients (subset 3: median = 24.41 pg/ml, range 0 to 411.2) than in SSc marker autoantibody-positive patients (subset 1 + subset 2 patients: median = 239.8 pg/ml, range 66.74 to 1,003 pg/ml; *P *= 0.0015) (Figure 3D). sIL-2Rα levels were lower than the 5th percentile of control values (25.7 pg/ml) in 11/17 (64.7%) subset 3 patients but not in any subset 1 or subset 2 patient (*P *= 0.0001).

The mean capillary number was >9/mm in 41 patients, 9 to 7/mm in 24 patients and 6 to 4/mm in six patients. No statistical difference was found among these three subgroups in the prevalence of each clinical, preclinical functional and laboratory abnormality investigated (data not shown). Finally, none of the investigated clinical (puffy fingers, arthritis), preclinical (DLCO/forced vital capacity reduction, E/A ratio inversion), or serological (SSc marker autoantibodies, ICTP, sE-selectin, sIL-2Rα) parameters was associated with the disease duration, as evaluated by RP onset.

## Discussion

To our knowledge, this is the first study to systematically explore clinical and preclinical organ involvement and circulating activation markers in early SSc patients subdivided into three subsets, depending on the presence of both marker autoantibodies and NVC scleroderma pattern or either of these features.

Firstly, we found that our early SSc patients, which were enrolled by two distinct clinical units, were similar in sex and age distribution and disease duration, but differed in terms of clinical features and serum levels of fibroblast, endothelial, and T-cell activation markers at presentation.

Given the different target patients of the two units, it is not surprising that a higher prevalence of SSc marker autoantibody-positive patients was enrolled by the Rheumatology Unit whereas a higher prevalence of RP patients presenting with puffy fingers was enrolled by the Angiology Unit. These differences are in line with results reported in patients with definite SSc by Walker and colleagues, who analyzed data entered into the European League Against Rheumatism (EULAR) Scleroderma Trials and Research database and found that differences among patients from various centers depend mainly on the clinical attitude of the respective medical team [16]. Notwithstanding this possible center-related recruitment bias, in our study early SSc patients with marker autoantibodies had a higher prevalence of impaired DLCO and higher circulating levels of ICTP irrespective of an NVC scleroderma pattern (megacapillaries and/or avascular areas). Similarly, early SSc patients with an NVC scleroderma pattern had a higher prevalence of puffy fingers and higher circulating levels of E-selectin irrespective of circulating marker autoantibodies, the two features being closely related each other. These associations have never previously been searched for. Moreover, four out of the 14 Rheumatology Unit patients with DLCO <80% of the predicted value already presented mild alterations of lung anatomy as detected by high-resolution computed tomography.

In this context, it is noteworthy that an impaired DLCO is considered the first alteration in SSc interstitial lung disease [14,17]. Increased ICTP levels have been reported in SSc patients [18] and suspected SSc patients (that is, patients not meeting the preliminary 1990 ACR criteria for SSc) [4]. Moreover, elevated ICTP levels have been found to correlate with modified Rodnan skin score and to be associated with a diffuse disease and a reduced pulmonary function [19]. Given these observations, it is conceivable that early SSc patients with SSc marker autoantibodies are at higher risk of developing fibrotic organ complications.

Puffy fingers and RP are considered the main clinical features of the undifferentiated connective tissue syndrome [20]. sE-selectin, which reflects endothelial activation, has been reported to be increased in patients with definite SSc [21]. Our study demonstrates that puffy fingers are detectable in early SSc patients, mainly in those with an NVC scleroderma pattern and increased sE-selectin levels. Serum IL-2Rα levels, which reflect the activation of the adaptive immune response through T-cell recruitment, have been found to be strongly associated with mortality and inversely correlated to disease duration in SSc patients with diffuse disease [22]. Intriguingly, we found that sIL-2Rα levels were strikingly lower in early SSc patients who were marker autoantibody-negative. This result could indicate a lack of T-cell recruitment in the peripheral blood of autoantibody-negative early SSc patients.

Taken together, the results of our study seem to suggest that the major cellular pathways involved in the pathogenesis of SSc, as evaluated by serum concentration of relevant activation markers, differ among early patients at presentation, in relation to autoantibody and microvascular status. Nevertheless, the asynchronous appearance of autoantibody positivity and capillaroscopic alterations has been reported in two recent papers. First, Englert and colleagues described the case of one patient in whom anti-Scl-70 positivity, associated with fatigue, weight loss and puffy fingers, predated the appearance of RP and the development of capillaroscopic abnormalities [23]. Second, Moinzadeh and colleagues reported the late appearance of serum autoantibodies in three patients with an NVC scleroderma pattern at admission [24]. Whether SSc marker autoantibody positivity or the presence of an NVC scleroderma pattern really do identify the dominating activation of a given cellular pathway at the disease onset in early SSc patients can only be determined by investigating circulating and tissue markers of cell activation contemporaneously.

Our study has some limitations. First, it might be affected by a differential verification bias because the patients were examined by clinicians with different medical expertise. To reduce this possible bias, however, capillaroscopic images and sera were made anonymous and centralized to the Rheumatology Unit. Capillaroscopic images were blindly evaluated by one investigator experienced in NVC (MI); sera were blindly analyzed in duplicate by one investigator experienced in immunoassays (SV). Moreover, the clinical (puffy fingers) and preclinical (DLCO and E/A ratio) parameters used to assess both series of patients are well defined and routinely applied in clinical practice. Second, given the small number of patients, our conclusions may be susceptible to be changed by future studies on large number of patients, such as the ongoing Very Early Diagnosis of Systemic Sclerosis (VEDOSS) project [25,26]. In addition, one must underline that using the new ACR/EULAR classification criteria presented at the ACR 2012 meeting [27,28], a number of our early SSc patients could already meet the criteria for SSc at presentation. Indeed, in a *post-hoc *analysis 10/71 (14.1%) patients met the new EULAR/ACR classification criteria for SSc and belonged to subset 1 (that is, patients with both SSc marker autoantibodies and capillaroscopic abnormalities). These patients met the new criteria because of the presence of puffy fingers along with SSc marker autoantibodies and the NVC scleroderma pattern. However, our major findings - that is, early SSc patients with SSc marker autoantibody have higher ICTP levels, regardless of the NVC pattern; and early SSc patients with an NVC scleroderma pattern have a higher prevalence of puffy fingers and higher circulating levels of E-selectin, regardless the autoantibody profile, the two features being closely related each other - did not differ significantly when we excluded these 10 patients from the analysis (see Additional files 1, 2, 3 and 4).

In conclusion, we found that early SSc patients show distinct clinical and preclinical features, and distinct circulating markers of cellular activation at presentation, according to their autoantibody and capillaroscopic status. Longitudinal large studies are required to verify whether the pathophysiological (cellular activation markers), functional (DLCO) and clinical (puffy fingers) differences in early SSc subsets that we detected in a small yet well characterized cohort really do translate into the development of different disease patterns over time.

## Abbreviations

ACR: American College of Rheumatology; DLCO: diffusing lung capacity for carbon monoxide; E/A ratio: early/(atrial) late ventricular filling velocity ratio; ELISA: enzyme-linked immunosorbent assay; EULAR: European League Against Rheumatism; ICTP: carboxyterminal telopeptide of type I collagen; NVC: nailfold videocapillaroscopy; RP: Raynaud's phenomenon; sE-selectin: soluble E-selectin; sIL-2Ra: soluble IL-2 receptor alpha; SSc: systemic sclerosis.

## Competing interests

The authors declare that they have no competing interests.

## Authors' contributions

GV conceived, designed and coordinated the study, and drafted and critically revised the manuscript. AM acquired clinical data, performed nailfold videocapillaroscopy and participated in the design of the study. GC acquired clinical data, performed the statistical analysis, and participated in the design of the study and in drafting the manuscript. SV performed all immunoassays, the statistical analysis, and the *post-hoc *analysis, and drafted and critically revised the manuscript. MI performed nailfold videocapillaroscopy and participated in the acquisition of data. FB performed autoantibody testing and routine biochemical analysis. CS and AC performed and analyzed lung function tests. DC and GMDM performed and analyzed B-mode echocardiography. SC and FV performed and analyzed chest X-ray scans. AS participated in the design of the study and critically revised the manuscript. All authors read and approved the final manuscript.

## Supplementary Material

Additional file 1 **Table S1 presenting epidemiologic, laboratory and capillaroscopic features in patients who did not meet EULAR/ACR classification criteria, and figure legends for Figures S1, S2, and S3**. Click here for file

Additional file 2 **Figure S1 showing the prevalence of puffy fingers, arthritis and NVC scleroderma pattern in patients who did not meet EULAR/ACR classification criteria**. Click here for file

Additional file 3 **Figure S2 showing the prevalence of DCLO impairment and autoantibody positivity in patients who did not meet EULAR/ACR classification criteria**. Click here for file

Additional file 4 **Figure S3 showing serum levels of ICTP, sE-selectin, sIL2Rαa and sE-selectin in patients who did not meet EULAR/ACR classification criteria**. Click here for file
