# Supplementary material for: Early systemic sclerosis: marker autoantibodies and videocapillaroscopy patterns are each associated with distinct clinical, functional and cellular activation markers
Source: Arthritis Res Ther. 2013 May 29;15(3):R63. doi: 10.1186/ar4236 (PMC4060381; doi:10.1186/ar4236)
Supplement: Additional file 1 — Table S1 presenting epidemiologic, laboratory and capillaroscopic features in patients who did not meet EULAR/ACR classification criteria, and figure legends for Figures S1, S2, and S3. [file ar4236-S1.DOCX]

**Table S1. Epidemiologic, laboratory and capillaroscopic features of the two early SSc series, excluding patients meeting the new EULAR/ACR classification criteria.**

| **Feature** | **Rheumatology Unit**  **(n = 41)** | **Angiology Unit**  **(n = 20)** | **P** |
| --- | --- | --- | --- |
| **Sex: F/M ratio** | 39/2 | 16/4 | ns |
| **Age; years (median; range)** | 41 (17-73) | 34 (16-61) | 0.04 |
| **RP duration years (median; range)** | 3 (0.5-24) | 3 (1-20) | ns |
| **Subset 1: Ab + NVC +** |  | | |
|  | 22 (53.7) | 0 | - |
| **Anti-Scl-70 ab +**  **ACA +**  **Anti-RNA polymerase III ab +**  **Megacapillaries only**  **Avascular areas ± megacapillaries** | 4 (18.2)  18 (81.8)  0  19 (86.4)  3 (13.6) | 0  0  0  0  0 |  |
|  |  | | |
| **Subset 2: Ab + NVC -** | 16 (35.6) | 0 | - |
| **AntiScl-70 ab +**  **ACA +**  **Anti-RNA polymerase III ab +** | 4 (25)  12 (75)  0 | 0  0  0 |  |
|  |  | | |
| **Subset 3: Ab - NVC +** | 3 (6.7) | 20 (76.9) | 0.0001 |
| **Megacapillaries only**  **Avascular areas ± megacapillaries** | 3 (100)  0 | 20 (100)  0 | -  - |

All data are expressed as numbers and percentages (in brackets), except where otherwise indicated

**Supplementary figure legend:**

**Supplementary figure 1.** Prevalence of puffy fingers (A) and arthritis (B) in the 61 early SSc patients from both centres who did not meet the new EULAR/ACR classification criteria for the disease, divided into three subsets according to the SSc marker autoantibody status and the NVC pattern: presence of both SSc marker autoantibodies and NVC scleroderma pattern (subset 1), presence of SSc marker autoantibodies only (subset 2), and presence of NVC scleroderma pattern only (subset 3). The prevalence of puffy fingers in subset 1 patients could not be evaluated, as the contemporary presence of these feature along with the SSc markers autoantibodies and the NVC scleroderma pattern allows the classification of patients according to the new EULAR/ACR criteria. Prevalence of NVC scleroderma pattern (C) in all patients from both centres divided according to the presence/absence of puffy fingers. Chi-Square test was applied for statistical analysis of data reported in A and B; Fisher’s exact test was applied for statistical analysis of data reported in C.

SSc = systemic sclerosis; NVC = nailfold videocapillaroscopy; n = number of patients

**Supplementary figure 2.** Prevalence of DLCO impairment (less than 80% of the predicted value) (A) in the 61 early SSc patients from both centres who did not meet the new EULAR/ACR classification criteria for the disease, divided into three subsets according to the SSc marker autoantibody status and the NVC pattern: presence of both SSc marker autoantibodies and NVC scleroderma pattern (subset 1), presence of SSc marker autoantibodies only (subset 2), and presence of NVC scleroderma pattern only (subset 3). Prevalence of general SSc marker autoantibody positivity (B) and prevalence of either ACA or other SSc marker autoantibody positivity (C) in all patients from both centres divided according to the presence/absence of DLCO impairment. Chi-Square test was applied for statistical analysis of data reported in A. Fisher’s exact test was applied for statistical analyses of data reported in B and C.

DLCO = diffusing lung capacity for CO; SSc = systemic sclerosis; NVC = nailfold videocapillaroscopy; Abs = autoantibodies; n = number of patients

**Supplementary figure 3.** Serum levels of fibroblast - ICTP (A), endothelial - sE-selectin (B), and T-cell - sIL2Rαa (D) activation markers in 25 controls affected by ostheoarthritis or fibromyalgia, and 41 early SSc patients who did not meet the new EULAR/ACR classification criteria for the disease, divided into three subsets according to the SSc marker autoantibody status and the NVC pattern: presence of both SSc marker autoantibodies and NVC scleroderma pattern (subset 1), presence of SSc marker autoantibodies only (subset 2), and presence of NVC scleroderma pattern only (subset 3). Serum levels of sE-selectin (C) in all patients from both centres divided according to the presence/absence of puffy fingers. Kruskal-Wallis and Mann-Whitney U test were applied for statistical analysis of data reported in A, B, and D. Mann-Whitney U test was applied for statistical analysis of data reported in C.

ICTP = carboxyterminal propeptide of collagen I, sE-selectin = soluble E-selectin; sIL2Rα = soluble IL-2-receptor-α; SSc = systemic sclerosis; NVC = nailfold videocapillaroscopy; n = number of patients
