# Supplementary material for: Early systemic sclerosis: marker autoantibodies and videocapillaroscopy patterns are each associated with distinct clinical, functional and cellular activation markers
Source: Arthritis Res Ther. 2013 May 29;15(3):R63. doi: 10.1186/ar4236 (PMC4060381; doi:10.1186/ar4236)
Supplement: Additional file 2 — Figure S1 showing the prevalence of puffy fingers, arthritis and NVC scleroderma pattern in patients who did not meet EULAR/ACR classification criteria. [file ar4236-S2.PPT]

## Slide 1
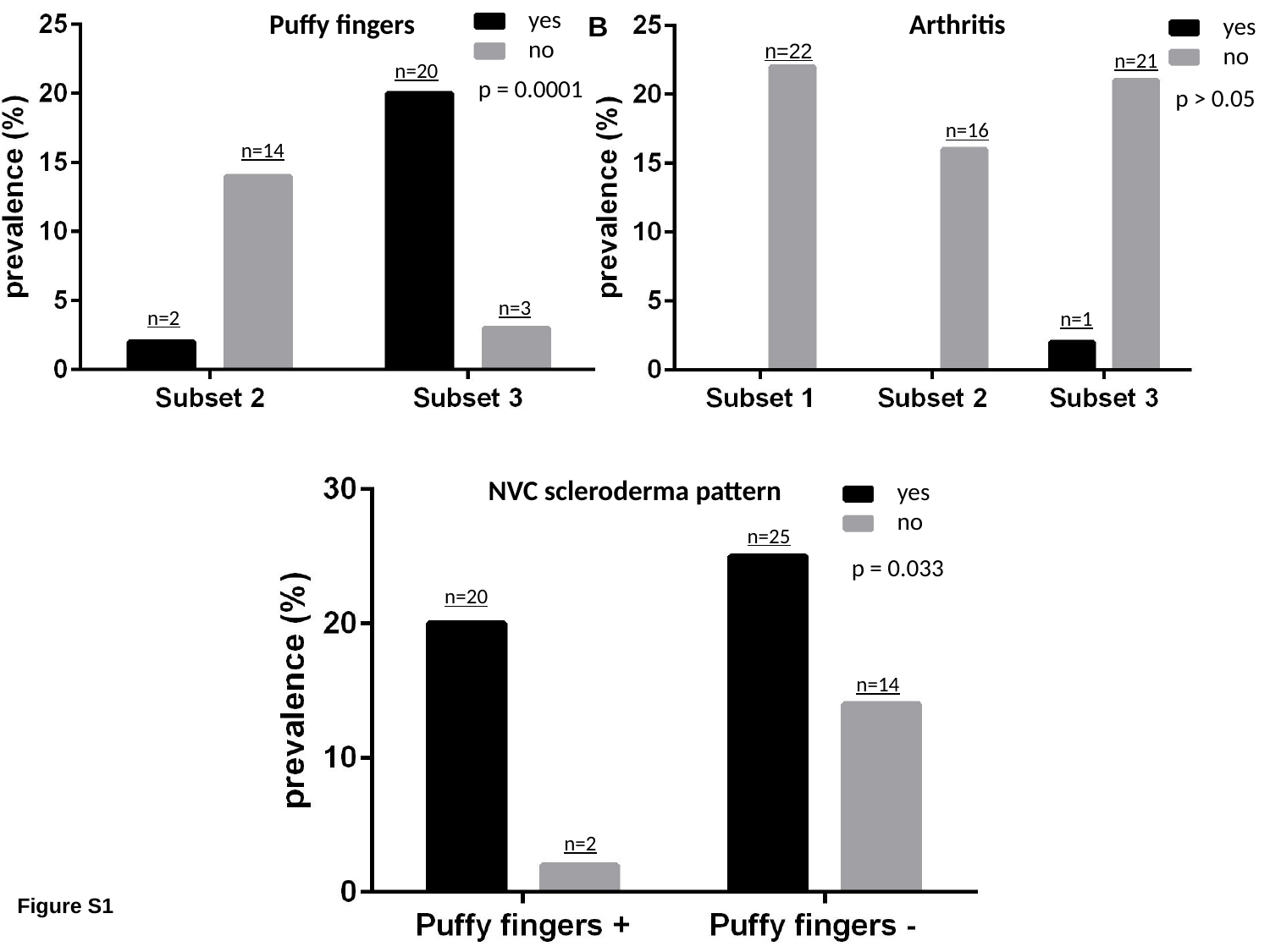

A
Puffy fingers
yes
no
n=20
p = 0.0001
n=14
n=3
n=2
Arthritis
B
yes
no
n=22
n=21
p > 0.05
n=16
n=1
NVC scleroderma pattern
yes
no
n=25
p = 0.033
n=20
n=14
n=2
Figure S1
C
