# Supplementary figures and images for: Early systemic sclerosis: marker autoantibodies and videocapillaroscopy patterns are each associated with distinct clinical, functional and cellular activation markers
Source: Arthritis Res Ther. 2013 May 29;15(3):R63. doi: 10.1186/ar4236 (PMC4060381; doi:10.1186/ar4236)

## Slide 1
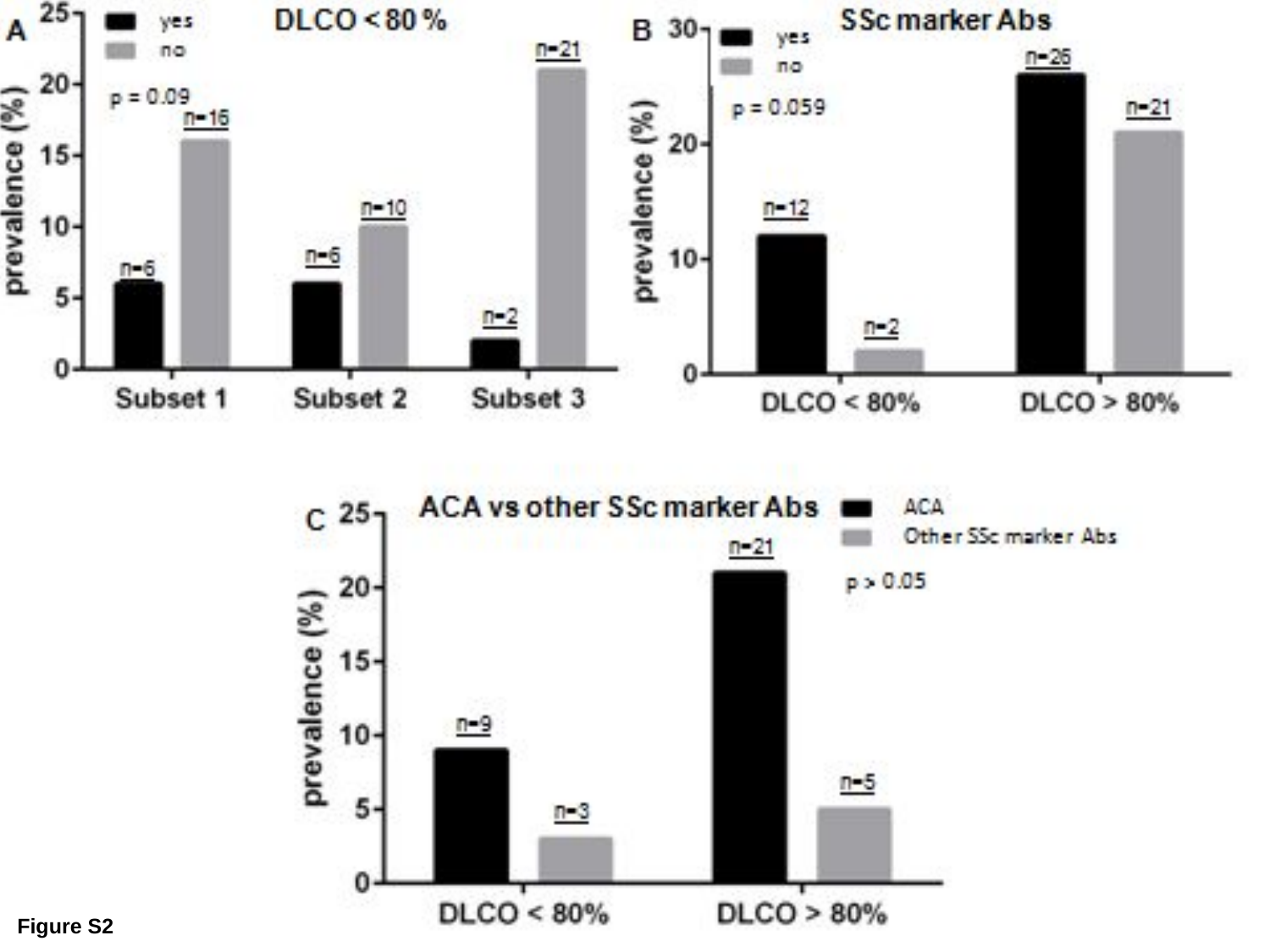

Figure S2

Supplement: Additional file 3 — Figure S2 showing the prevalence of DCLO impairment and autoantibody positivity in patients who did not meet EULAR/ACR classification criteria. [file ar4236-S3.PPT]

## Slide 1
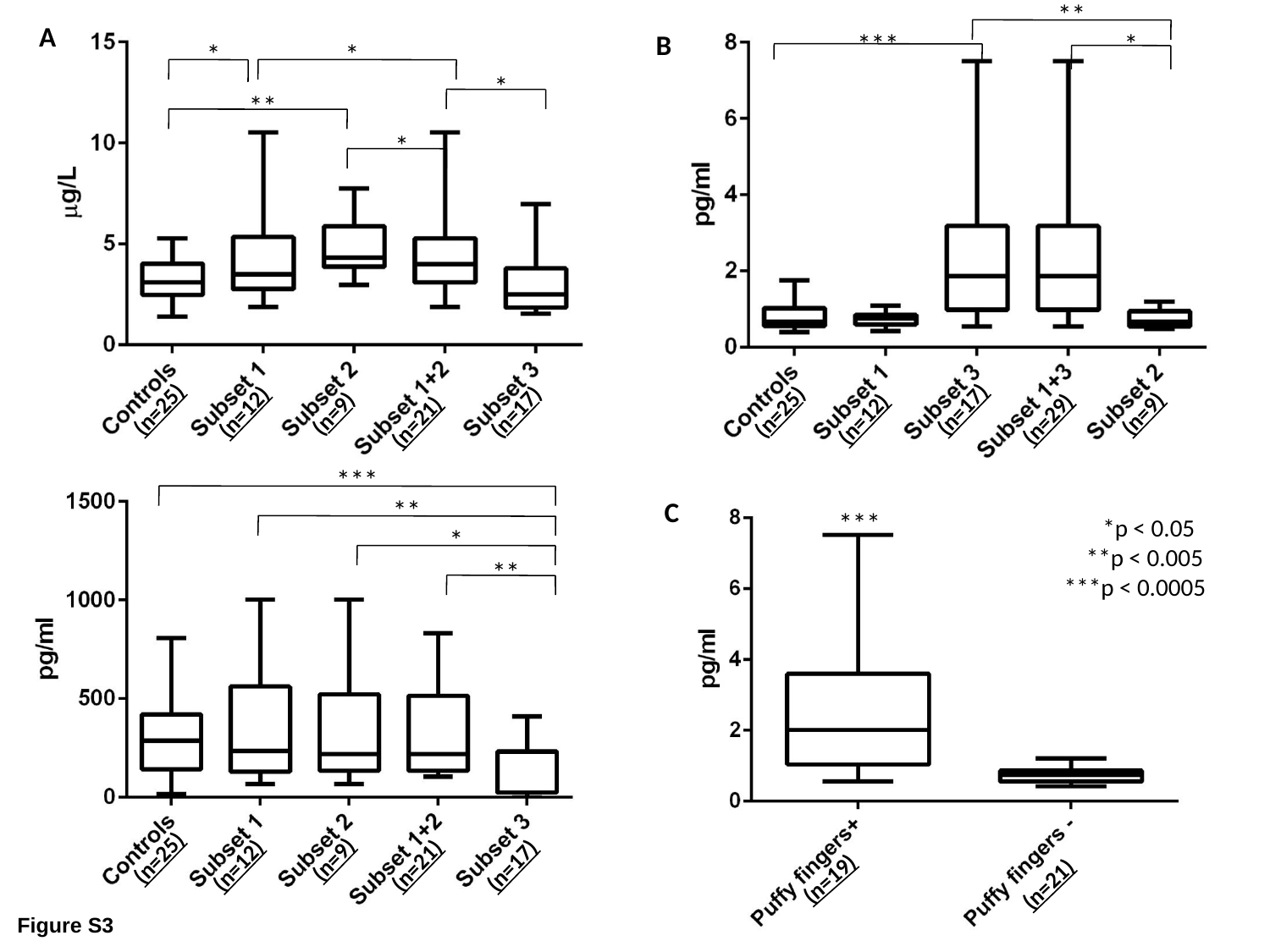

A
(n=25)
(n=9)
(n=12)
(n=17)
(n=21)
B
(n=17)
(n=9)
(n=25)
(n=12)
(n=29)
C
(n=19)
(n=21)
***
**
***
*
*
*
*
**
*
***
**
*
**
(n=9)
(n=25)
(n=12)
(n=21)
(n=17)
D
 *p < 0.05
 **p < 0.005
***p < 0.0005
Figure S3

Supplement: Additional file 4 — Figure S3 showing serum levels of ICTP, sE-selectin, sIL2Rαa and sE-selectin in patients who did not meet EULAR/ACR classification criteria. [file ar4236-S4.PPT]
